# The mediating role of sexually selected traits in the association of androgynous tendencies with lower sexual activeness among Chinese youths

**DOI:** 10.3389/fpsyg.2022.1011467

**Published:** 2022-10-06

**Authors:** Shuangyu Zhao, Fuyu Guo, Jia Yi Hee, Kun Tang

**Affiliations:** ^1^Vanke School of Public Health, Tsinghua University, Beijing, China; ^2^Department of Epidemiology, T.H. Chan School of Public Health, Harvard University, Boston, MA, United States

**Keywords:** sexual selection, gender-role conformity, androgynous tendencies, sexual activeness, low fertility rate

## Abstract

Androgynous tendencies and persistently low fertility rates have been observed in many countries, causing major social concerns. The theory of sexual selection suggests a possible mechanism between androgyny and decreased sexual activeness, as masculinity and femininity constitute an important aspect of reproductive strategies. This theory has also been proven by evolutionary and societal evidence. Therefore, we investigate the association between masculinity and femininity with sexual activeness, as well as the influence of gender-role conformity on the frequency of sexual intercourse through sexually selected traits among 42,492 Chinese youths. Sexual activeness was measured using sexual attitudes, experience, behaviors, and pleasure. Mediation analysis was employed to investigate the effects of sexually selected traits on the association between masculinity and femininity with sexual activeness, and gender-role conformity with the frequency of sexual intercourse. Low sexual activeness was found to be associated with low gender-role conformity. Our findings also suggest that physical attractiveness, sexual motivation, and interpersonal relationships may mediate the association between sexual activeness and gender-role conformity, supporting the males-compete/females-choose model.

## Introduction

Sexual selection was defined in the book by Charles Darwin, the Descent of Man (1871), as “the advantage which certain individuals have over others of the same sex and species solely in respect of reproduction”(Darwin, 2008). Based on the theory of sexual selection, ecological traits including masculinity and femininity (also referred to as gender roles), are considered to constitute a portion of human reproductive strategies (Cornwell et al., 2006; Kruger, 2006). The theory that high masculinity and femininity traits serve as indicators of high sexual activeness is supported by a series of studies on evolution, anthropology, physiology, and sociology. Masculinity in males was observed to shape physical characteristics such as jaw size, muscularity, and midface robustness (Marcinkowska et al., 2019), to reflect certain traits such as mate quality, paternal investment, and competitiveness (Bribiescas et al., 2012), while facial femininity in females was also observed to be associated with fecundability (Little et al., 2014). Studies have demonstrated that prenatal exposure to high levels of testosterone plays a significant role in the construction of adult masculinity (Imperato-McGinley et al., 1979) and increases sexual activeness in males (Halpern et al., 1998). However, high levels of testosterone in females result in decreased sexual activeness (Law Smith et al., 2012; Das and Sawin, 2016; Niu and Zheng, 2020). Additionally, certain moral and quasi-moral traits relevant to femininity in females, including kindness and agreeableness (Eisenberg et al., 2001), have been sociologically and biologically demonstrated to increase sexual attractiveness (Zietsch et al., 2008).

Interestingly, an increasing trend of androgynous tendencies has been observed among Chinese youths over the past few decades. A survey of Chinese college students conducted in 1998 reported that while 32.3% of male students viewed themselves as masculine and 69.6% female students viewed themselves as feminine, 23.1% and 15.4% of male and female students, respectively, viewed themselves as androgynous (Shaomei, 1998). However, a more recent survey on 5,286 Chinese college students in 2011 reported that the proportion of male and female students who viewed themselves as masculine and feminine has decreased to 25.0% and 27.3%, respectively, while the proportion of male and female students who viewed themselves as androgynous has increased to 33.01% and 29.58%, respectively (Dianzhi, 2011). During the same period, the total fertility rate (TFR) in China declined below replacement levels from 2.7 in 1988 (Abbafati et al., 2020) to 1.7 in 2021 (Searchinger et al., 2013; Shen, 2021), leading to major economic and social concerns. As androgyny may impact sexual behaviors which correlates with fertility, it is therefore of great social significance to evaluate the potential roles of androgynous tendency on sexual activeness among youths of reproductive age.

At present, population-based studies on the relationship between gender-role conformity and sexual activeness or fertility decline are limited. Theories on the declination of fertility suggest that higher education, welfare institutions (Freedman, 1979), and greater stress of living (Liu and Raftery, 2020) contribute to fewer births. However, these studies often draw largely on various sociological theories, while disregarding the biological nature of human sexuality (Duckworth and Trautner, 2019). Furthermore, these studies typically focus on sexual minorities or individuals with sexually transmitted infections and tend to focus only on masculinity and not femininity (Little et al., 2011). Therefore, the present study aims to investigate the association between gender-role conformity and sexual activeness among male and female heterosexual Chinese youths. Mediation analysis was also employed to investigate the effect of sexually selected traits in the associations.

## Materials and methods

### Data source and participants

Our study utilized data from the 2019 National College Student Survey on Sexual and Reproductive Health, an internet-based self-administered survey conducted from November 2019 to February 2020. The study which aims to assess the sexual and reproductive health (SRH) of Chinese students, was commissioned by the China Family Planning Association (CFPA). The development of the questionnaire and study design has been previously described in detail (Zou et al., 2021). The questionnaire contained 92 questions categorized into four sections: SRH knowledge and attitudes, intimacy and sexual experience, individual health status, and socioeconomic and demographic characteristics.

A multistage sampling approach was utilized, and 1,764 higher education institutions, including key universities, ordinary universities, and vocational colleges, were selected after balancing the types of educational institutes. A total of 55,757 students responded. For the purposes of this study, only respondents who (1) provided informed consent, (2) answered all questions and passed the consistency checks and logic verification, (3) were aged between 15 and 25 years old, and (4) were undergraduate students who self-reported their gender orientation as heterosexual, were included. A total of 42,492 university or vocational college students were included in the final analyses. This study has been approved by the Institutional Review Board of Tsinghua University (IRB No. 20190083).

### Exposure

The main exposure of interest was participants’ self-rated conformity to their gender roles (masculinity for males and femininity for females), which was assessed using the question “I consider myself as conforming to traditional gender roles” adopted from the traditional masculinity-femininity (TMF) scale (Kachel et al., 2016). Response options ranged from 1 (strongly disagree) to 7 (strongly agree). Self-rated gender-role conformity scores were standardized by subtraction of the mean value and division by the standard deviation (SD). Gender-role conformity scores were categorized into low and high using the median value as a cutoff point (six for males and five for females).

### Outcomes

The main outcome of interest was sexual activeness measured using sexual attitudes, experience, behaviors, and satisfaction. Attitudes toward sexual behaviors were assessed using the following questions: “If one has premarital sex, he or she will have a more satisfied marriage,” and “You have ever felt guilty for being sexually active.” Response options were “strongly disagree,” “disagree,” “not sure,” “agree,” and “strongly agree.” The options “agree” and “strongly agree” were categorized as positive attitudes, while the other options were categorized as neutral/negative attitudes.

Sexual experience was assessed by two aspects: having had intimate partners (i.e., girlfriends for males and boyfriends for females) and the age of first penetrative sexual intercourse. The age of first penetrative sexual intercourse was dichotomized into two categories: before and after 16 years of age. First penetrative sexual intercourse before 16 years of age was considered to be early first penetrative sexual intercourse.

Sexual behaviors were assessed by two aspects: having ever had penetrative sexual intercourse (anal or vaginal) and the frequency of sexual intercourse in the past year. The frequency of sexual intercourse was measured based on how frequently the participants had sexual intercourse. Response options were “never had sexual intercourse,” “have not had sexual intercourse in recent years,” “have sexual intercourse several times per year,” “have sexual intercourse several times per month,” “have sexual intercourse three to five times per week,” “have sexual intercourse one to two times a week,” and “have sexual intercourse nearly every day.” Those who selected “have sexual intercourse three to five times per week,” “have sexual intercourse one to two times per week,” and “have sexual intercourse nearly every day” were categorized as having frequent sexual intercourse.

Sexual satisfaction was assessed using the following questions: “Have you ever had an orgasm?” and “Do you feel satisfied with the sex you are having currently?.” Responses “have ever” to the first question were considered to indicate sexual satisfaction, while responses “have never” or “I do not know what an orgasm is” were considered to indicate no sexual satisfaction. Responses “very satisfied” or “satisfied” to the second question were considered to indicate sexual satisfaction, while responses “neutral,” “not satisfied,” or “strongly not satisfied” were considered to indicate no sexual satisfaction.

### Other covariates

Data on sex, age, ethnicity, hometown region, school type, average monthly expenditure, having had received sexual education at school, self-rated parent–child relationship, parental highest educational attainments, having had parent–child discussions relevant to sexual behaviors, and tobacco/alcohol consumption were also collected. Age and self-rated parent–child relationship scores were analyzed as continuous variables, while the remaining variables were analyzed as categorical variables. Self-rated parent–child relationship scores ranged from 0 (terrible) to 10 (good).

### Statistical analysis

Continuous variables were reported as mean **±** SD, while categorical variables were described as proportions (percentages). Categorical variables were compared using Pearson’s *χ*^2^ test, and continuous variables were compared using *t-*test. Multivariable logistic regression was used to assess the association between gender-role conformity scores and sexual attitudes, experience, behaviors, and satisfaction. The results were reported as odds ratio (OR) and 95% confidence intervals (95% CIs). The models were adjusted for age, ethnicity, school type, average monthly expenditure, self-rated parent–child relationship, having had received sexual education at school, parents’ highest educational attainments, and tobacco and alcohol assumption. Analyses for “early age of first penetrative sexual intercourse,” “having had an orgasm,” and “current sexual satisfaction” were limited to respondents who reported having had sexual intercourse.

### Mediation analysis

Mediator variables were classified into individual-and interpersonal-level factors. Individual-level factors included self-perceived physical attractiveness and sexual motivation, while interpersonal-level factors included self-rated interpersonal relationships. Self-perceived physical attractiveness was assessed using a 10 point scale ranging from 0 (terrible) to 10 (excellent), with higher scores implying higher satisfaction with appearance. Sexual motivation was assessed using the following question: “Do you want to have a girlfriend/boyfriend?” Response options included “I really want a girlfriend/boyfriend,” “I want a girlfriend/boyfriend,” “I am unsure,” “I do not want a girlfriend/boyfriend,” and “I really do not want a girlfriend/boyfriend.” Responses “I really want a girlfriend/boyfriend” or “I want a girlfriend/boyfriend” were considered to indicate high sexual motivation. Self-rated interpersonal relationships were assessed using the following question: “Do you have many friends?” Response options ranged from 1 (nearly have no friend) to 10 (have many friends).

**Figure 1:**
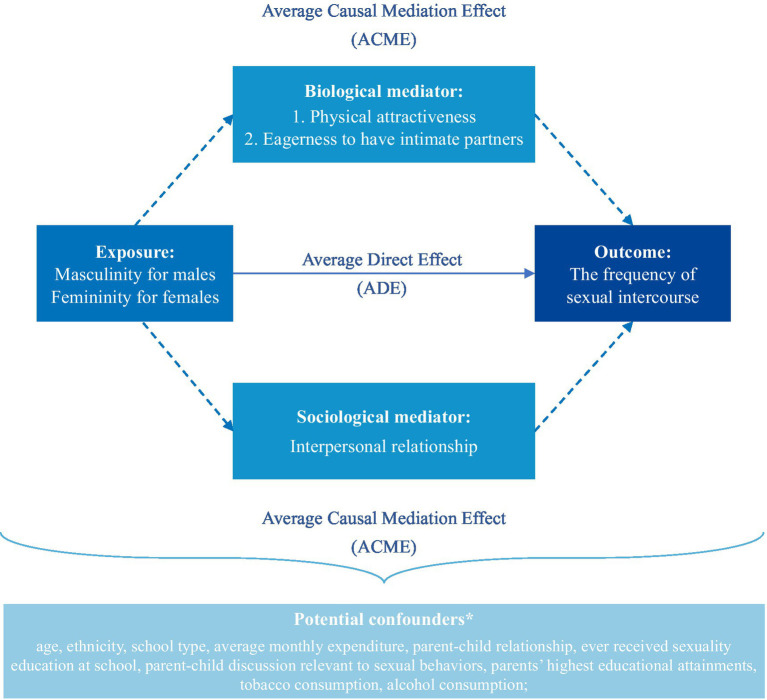
Conceptual diagram.

First, logistic regression was used to investigate the association between individual- and interpersonal-level mediators with frequent sexual intercourse (Supplementary Table S1). Second, mediation modeling frameworks were constructed (Figure 1), with A representing exposure, M representing mediators, Y representing outcome, and C representing a set of confounders. The total effect (TE) of gender roles on frequent sexual intercourse could be present through two separate pathways: (1) directly between gender roles and frequent sexual intercourse, measured using the average direct effect (ADE), or (2) through biological and societal mediators, measured using the average causal mediation effect (ACME).

In the framework, $T_{i}$ denoted continuous exposure representing the different SDs of the gender-role conformity score. $Y_{i}\left(t\right)$ was used to characterize the binary outcome variable under exposure status t , which was equal to 1 if respondent i reported frequent sexual intercourse (i.e., $Y_{i}\left(1\right)$ estimated respondent $i^{’}$ s sexual intercourse status, whose gender-role conformity score was 1 SD). In addition, $M_{i}\left(t\right)$ was used to measure mediators under exposure status t , and $X_{i}$ denoted the abovementioned set of confounders. If the mediator was a continuous variable (self-perceived physical attractiveness and self-rated interpersonal relationship), we used formula (1) to measure the association between mediators and exposure and formula (2) to characterize the relationship between outcome and exposure, considering the mediator effect:


(1) $$\;M_{i}=\alpha_{2}+\beta_{2}T_{i}+\xi_{2}^{⊺}X_{i}+\epsilon_{2i}$$

(2) $$\;Y_{i}=1\;\left\{Y_{i}^{*}>0\right\}\;\mathrm{where}\;Y_{i}^{*}=\alpha_{3}+\beta_{3}T_{i}+\gamma M_{i}+\xi_{3}^{⊺}X_{i}+\epsilon_{3i}$$

Considering that there might have been an exposure-mediator interaction, formula (2) was rewritten as formula (3):


(3) $$\begin{array}{c} \;Y_{i}=1\;\left\{Y_{i}^{*}>0\right\}\;\\ \mathrm{where}\;Y_{i}^{*}=\alpha_{3}+\beta_{3}T_{i}+\gamma M_{i}+\kappa T_{i}M_{i}+\xi_{3}^{⊺}X_{i}+\epsilon_{3i} \end{array}$$

ACME ( $\delta_{i})$ was defined as the difference between the potential outcome when the exposure value was fixed and the mediator level intervening from $M_{i}\left(t_{0}\right)\;$ to $M_{i}\left(t_{1}\right)$ . ADE ( $\zeta_{i}$ ) estimated the difference between potential outcome when the value of mediator was fixed and the exposure level intervening from $t_{0}\;$ to $t_{1}$ . TE ( $\tau_{i}$ ) was the sum of the ACME and ADE:


(4) $$\;\delta_{i}\left(t\right)\equiv Y_{i}\left(t,M_{i}\left(t_{1}\right)\right)-Y_{i}\left(t,M_{i}\left(t_{0}\right)\right)$$

(5) $$\;\zeta_{i}\left(t\right)\equiv Y_{i}\left(t_{1},M_{i}\left(t\right)\right)-Y_{i}\left(t_{0},M_{i}\left(t\right)\right)$$

(6) $$\begin{array}{c} \;\tau_{i}\equiv Y_{i}\left(t_{1},M_{i}\left(t_{1}\right)\right)-Y_{i}\left(t_{0},M_{i}\left(t_{0}\right)\right)\\ =\frac{1}{2}\sum_{t=t_{0}}^{t=t_{1}}\left\{\delta_{i}\left(t\right)+\zeta_{i}\left(t\right)\right\} \end{array}$$

The PM was defined as follows:


(7) $$\;PM=\delta_{i}\left(t\right)/\zeta_{i}\left(t\right)$$

Point estimates for the ACME, ADE, TE, and proportion of mediation (PM), as well as their respective 95% CIs, were estimated with 1,000 bootstrap resamples. The estimated coefficients are linked to the probability scale, rather than to that obtained from the logistic regression model, with its exponentiation obtaining the OR. The formulas were adjusted for binary variables. All formulas were deduced mathematically by Dustin Tingley (Imai et al., 2010).

Main analyses were performed using Stata SE™ version 15.1 (College Station, Texas, United States). Causal analyses were performed using R version 4.1.1.

## Results

### Differences in sample characteristics between high gender-role conformity and androgyny groups

The sociodemographic characteristics of male and female students are presented in Table 1. A total of 14,316 males and 28,176 females were included in the study. Among males with high gender-role conformity, 78.88% resided in urban areas (androgyny: 74.88%), 28.90% have an average monthly expenditure above 2,000 yuan (androgyny: 23.71%), 26.92% have had parent–child discussions on sexual behaviors (low: 22.93%), and 54.87% have received sexual education at school (androgyny: 50.38%). Males with high gender-role conformity also reported better father–child relationship (high: 8.01 ± 2.11 vs. androgyny: 7.32 ± 2.17, *p* < 0.0001) and mother–child relationship (high: 8.49 ± 1.76 vs. androgyny: 7.83 ± 2.00, *p* < 0.0001) compared to males with low gender-role conformity.

**Table 1:** Sociodemographic characteristics of male and female students stratified by level of gender-role conformity.

Characteristics	Male		Value of *p*	Female		Value of *p*
	*N =* 14,316			*N =* 28,176		
	Low gender-role conformity (androgyny)	High gender-role conformity		Low gender-role conformity (androgyny)	High gender-role conformity	
	*N* = 6,262	*N* = 8,054		*N* = 10,782	*N* = 17,394	
	*n* (%)	*n* (%)		*n* (%)	*n* (%)	
Age (in years)^*^, mean ± standard deviation (SD)	19.70 ± 1.50	19.81 ± 1.50	<0.0001	19.69 ± 1.53	19.82 ± 1.52	<0.0001
**Ethnicity**			0.728			0.619
Han	5,735 (91.58)	7,363 (91.42)		9,708 (90.04)	15,693 (90.22)	
Minority	527 (8.42)	691 (8.58)		1,074 (9.96)	1,701 (9.78)	
**Hometown region**			<0.0001			<0.0001
Rural	1,573 (25.12)	1,701 (21.12)		2,809 (26.05)	3,841 (22.08)	
Urban/suburban	4,689 (74.88)	6,353 (78.88)		7,973 (73.95)	13,553 (77.92)	
**School type**			<0.0001			<0.0001
College	2,504 (39.99)	2,871 (35.65)		3,463 (32.12)	4,892 (28.12)	
University	3,758 (60.01)	5,183 (64.35)		7,319 (67.88)	12,502 (71.88)	
**Average monthly expenditure (RMB)**		<0.0001			<0.0001
0–999	1,020 (16.29)	1,012 (12.57)		1,860 (17.25)	2,681 (15.41)	
1,000–1,999	3,757 (60.00)	4,714 (58.53)		6,335 (58.76)	10,209 (58.69)	
≥2,000	1,485 (23.71)	2,328 (28.90)		2,587 (23.99)	4,504 (25.89)	
**Ever received sexual education at school**	3,155 (50.38)	4,419 (54.87)	<0.0001	6,266 (58.12)	10.597 (60.92)	<0.0001
Self-rated parent–child relationship score (0–10)^*^, mean ± SD
Paternal	7.32 ± 2.17	8.01 ± 2.11	<0.0001	6.86 ± 2.39	7.39 ± 2.23	<0.0001
Maternal	7.83 ± 2.00	8.49 ± 1.76	<0.0001	7.47 ± 2.17	7.99 ± 1.92	<0.0001
**Parental highest educational attainment**		<0.0001			<0.0001
Primary school and below	834 (13.32)	886 (11.00)		1,486 (13.78)	2,128 (12.23)	
Middle school	2,461 (39.30)	2,904 (36.06)		4,342 (40.27)	6,697 (38.50)	
High school	1,593 (25.44)	2,219 (27.55)		2,715 (25.18)	4,619 (26.56)	
College and above	1,374 (21.94)	2,045 (25.39)		2,239 (20.77)	3,950 (22.71)	
**Parent–child discussion relevant to sexual behaviors**	<0.0001			<0.0001	
Never	4,826 (77.07)	5,886 (73.08)		7,872 (73.01)	12,042 (69.23)	
Ever	1,436 (22.93)	2,168 (26.92)		2,910 (26.99)	5,352 (30.77)	
**Tobacco use**			<0.0001			<0.0001
Ever	1,447 (23.11)	2,193 (27.23)		750 (6.96)	945 (5.43)	
Never	4,815 (76.89)	5,816 (72.77)		10,032 (93.04)	16,449 (94.57)	
**Alcohol consumption**			<0.0001			<0.0001
Ever	3,212 (51.29)	4,630 (57.49)		3,216 (29.83)	4,820 (27.71)	
Never	3,050 (48.71)	3,424 (42.51)		7,566 (70.17)	12,574 (72.29)	
Self-rated masculinity/femininity score (1–7)^*^, mean ± SD	4.35 ± 0.94	6.49 ± 0.50	<0.0001	3.28 ± 0.82	5.73 ± 0.78	<0.0001

* Uses *t*-test; others use χ2 analyses. SD, standard deviation.

Among females with high gender-role conformity, 77.92% resided in urban areas (androgyny: 73.95%), 25.89% have an average monthly expenditure above 2,000 yuan (androgyny: 23.99%), 30.77% have had parent–child discussions on sexual behaviors (androgyny: 26.99%), and 60.92% have received sexual education at school (androgyny: 58.12%). Females with high gender-role conformity also reported better father–child relationship (high: 7.39 ± 2.23 vs. androgyny: 6.86 ± 2.39, *p* < 0.0001) and mother–child relationship (high: 7.99 ± 1.92 vs. androgyny: 7.47 ± 2.17, *p* < 0.0001) compared to females with low gender-role conformity.

Males with high gender-role conformity report significantly higher tobacco use (high: 27.23% vs. androgyny: 23.11%, *p* < 0.0001) and alcohol consumption (high: 57.49% vs. androgyny: 51.29%, *p* < 0.0001), while females with high gender-role conformity report significantly lower tobacco use (high: 5.43% vs. androgyny: 6.96%) and alcohol consumption (high: 27.71% vs. androgyny: 29.83%, *p* < 0.0001), compared to males and females with low gender-role conformity.

### Distribution of gender-role conformity among males and females

**Figure 2:**
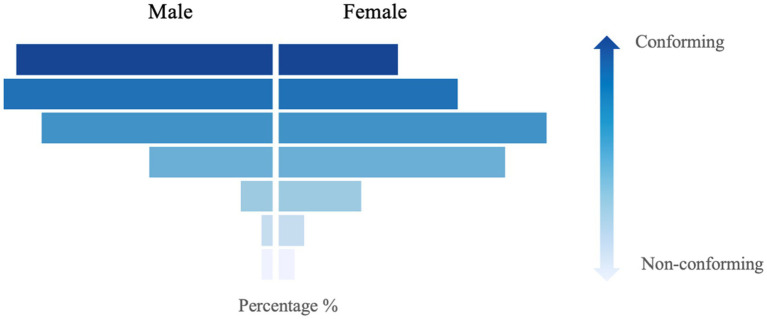
The distribution of gender-role conformity among male and female youths.

The distribution of gender-role conformity among males and females is presented in Figure 2. Males on average have a gender-role conformity score of 5.55 ± 1.28 on a scale of 7. There was a skewed distribution of gender-role conformity scores among males, with the majority reporting high masculinity conformity (56.25% reported scores of 6 points and above). On average, the gender-role conformity score was 6.49 ± 0.50 in the high masculinity group and 4.35 ± 0.94 in the androgynous group.

Females on average have a gender-role conformity score of 4.87 ± 1.36 on a scale of 7. In contrast to males, there was a normal distribution of gender-role conformity scores among females, with the majority reporting moderate femininity conformity score (53.91% reporting a score between 4 and 5), showing greater androgynous characteristics. On average, the gender-role conformity score was 5.73 ± 0.78 in the high femininity group and 3.28 ± 0.82 in the androgynous group.

### Association between androgynous tendency and sexual activeness

The association between gender-role conformity score and sexual activeness is presented in Table 2. Participants with high gender-role conformity were found to have more liberal attitudes toward sexual intercourse, were more likely to have intimate partners, were more likely to be sexually satisfied, and were more likely to exhibit more frequent sexual behaviors, compared to androgynous participants. Both males and females with high gender-role conformity were significantly more likely to agree with premarital sexual intercourse (male: OR 1.05, 95% CI 1.01–1.09, female: OR 1.06, 95% CI 1.03–1.09), have an intimate heterosexual partner (male: OR 1.18, 95% CI 1.13–1.22, female: OR 1.21, 95% CI 1.18–1.24), have had penetrative sexual intercourse (male: OR 1.12, 95% CI 1.07–1.17, female: OR 1.23, 95% CI 1.18–1.27), have more frequent sexual intercourse (male: OR 1.10, 95% CI 1.05–1.15, female: OR 1.20, 95% CI 1.15–1.24), and be sexually satisfied (male: OR 1.38, 95% CI 1.23–1.54, female: OR 1.15, 95% CI 1.08–1.23). Both males and females with high gender-role conformity scores were also significantly less likely to feel guilty for being sexually active (male: OR 0.85, 95% CI 0.82–0.89, female: OR 0.95, 95% CI 0.92–0.98), while males with high masculinity conformity scores were significantly more likely to have had an orgasm (OR 1.19, 95% CI 1.08–1.30). However, this was not significant in females with high femininity conformity scores (OR 1.05, 95% CI 0.99–1.11).

**Table 2:** The association between androgynous tendency and sexual activeness among males and females.

Variables	Sex^a^		Male	Female
**Openness toward sexual behaviors**		
Agree with premarital sex	1.05 (1.01–1.09)^*^	1.06 (1.03–1.09)^***^
Ever feel guilty for being sexually active	0.85 (0.82–0.89)^*^	0.95 (0.92–0.98)^**^
**Sexual experience**		
Have had a girlfriend/boyfriend	1.18 (1.13–1.22)^***^	1.21 (1.18–1.24)^***^
Early age of first penetrative sexual intercourse (before 16 years of age)^b^	1.09 (0.97–1.20)	1.05 (0.95–1.15)
**Sexual behaviors**		
Have had penetrative sexual intercourse (anal/vaginal)	1.12 (1.07–1.17)^***^	1.23 (1.18–1.27)^***^
Frequent sexual intercourse (more than once per week)	1.10 (1.05–1.15)^***^	1.20 (1.15–1.24)^***^
**Sexual pleasure**		
Have experienced an orgasm^b^	1.19 (1.08–1.30)^***^	1.05 (0.99–1.11)
Current sexual satisfaction^b^	1.38 (1.23–1.54)^***^	1.15 (1.08–1.23)^***^

The standardized gender-role conformity score is considered a continuous variable in all analyses, and the estimated odds ratio (OR) represents the change in odds followed by a 1-unit decrease in SD.

a Adjusted for age, region, ethnicity, school type, average monthly expenditure, self-rated parent–child relationship, if he or she has ever received sexual education at school, if he or she has ever had parent–child discussions relevant to sexual behaviors, parents’ highest educational attainments, tobacco consumption, and alcohol consumption.

b Analyze for individuals who have had sexual intercourse. n = 3,703 for males, and n = 5,046 for females.

* *p* < 0.05;

** *p* < 0.01;

*** *p* < 0.001.

### Roles of individual- and interpersonal-level mediators

The TE, ADE, ACME, and PM of the association between gender-role conformity and frequent sexual intercourse are presented in Table 3. The ACME and ADE of individual- and interpersonal-level factors were significant, indicating mediating effects of physical attractiveness, sexual motivation, and interpersonal relationships on the association between gender-role conformity and sexual activeness, regardless of sex. Exposure-mediator interactions were observed between physical attractiveness, sexual motivation, and interpersonal relationships with gender-role conformity scores in females, as well as between physical attractiveness with gender-role conformity scores in males (Supplementary Table S2).

**Table 3:** The mediation effect of sexually selected traits between gender-role conformity score and sexual activeness.

	TE	ACME (average)	ADE (average)	PM (average)
**Individual-level factors**				
Self-perceived physical attractiveness (0–10)				
Male	0.0197 (0.0111, 0.0268)^**^	0.0084 (0.0039, 0.0128)^**^	0.0113 (0.0055, 0.0159)^***^	42.48% (27.04, 58.89%)^**^
Female	0.0200 (0.0171, 0.0223)^***^	0.0057 (0.0039, 0.0074)^***^	0.0143 (0.0122, 0.0162)^***^	28.55% (21.67, 35.07%)^***^
**Sexual motivation**				
Male	0.0112 (0.0060, 0.0156)^**^	0.0016 (0.0001, 0.0031)^*^	0.0096 (0.0046, 0.0141)^**^	13.83% (0.45, 33.77%)^*^
Female	0.0126 (0.0096, 0.0155)^***^	0.0061 (0.0044, 0.0044)^***^	0.0065 (0.0034, 0.0090)^***^	47.99% (36.73, 65.04%)^***^
**Interpersonal-level factors**				
Self-rated interpersonal relationship (0–10)				
Male	0.0114 (0.0063, 0.0160)^***^	0.0048 (0.0031, 0.0067)^***^	0.0066 (0.0007, 0.0116)^*^	41.66% (23.71, 89.54%)^***^
Female	0.0196 (0.0174, 0.0216)^***^	0.0034 (0.0020, 0.0048)^***^	0.0162 (0.0143, 0.0178)^***^	17.23% (10.74, 23.20%)^***^

The standardized gender-role conformity score is considered a continuous variable in all analyses. All models are adjusted for age, region, ethnicity, school type, average monthly expenditure, self-rated parent–child relationship, if he or she has ever received sexual education at school, if he or she has had parent–child discussions relevant to sexual behaviors, parents’ highest educational attainments, tobacco consumption, and alcohol consumption. *ACME = average causal mediation effect,* ADE, average direct effect; PM, proportion of mediation.

a Analyze for individuals who have never had sexual intercourse. *n* = 10,613 for males, and *n* = 23,130 for females.

* *p* < 0.05;

** *p* < 0.01;

*** *p* < 0.001.

Among males, the PM by sexual motivation was 13.83% (95% CI: 0.45%–33.77%), by physical attractiveness, 42.48% (95% CI: 27.04%–58.89%), and by interpersonal relationship, 41.66% (95% CI: 23.71%–89.54%). Among females, the PM by sexual motivation was 47.99% (95% CI: 36.73%–65.04%), by physical attractiveness, 28.55% (95% CI: 21.67%–35.07%), and by interpersonal relationships, 17.23% (95% CI: 10.74%–23.20%).

## Discussion

Our findings suggest that heterosexual youths with low gender-role conformity, or androgyny, were less sexually active, and among those who had penetrative sexual intercourse, androgynous tendency was significantly associated with decreased sexual satisfaction. Our findings also suggest that physical attractiveness, sexual motivation, and interpersonal relationships may play a mediating role, indicating that sexually selected traits may be an important underlying mechanism in this pathway.

Our finding of a positive association between gender-role conformity and high sexual activeness is consistent with existing evidence. Previous studies showed that males with more masculine traits, such as certain facial characteristics, were more sexually active (Lidborg et al., 2022), while females with more feminine traits have more sexual partners. According to the theory of sexual selection, masculinity and femininity are regarded as part of human reproductive strategies. Hence, individuals with high masculinity and femininity are considered more “prepared” for reproduction. These factors may contribute to more liberal sexual attitudes and more aggressive sexual behaviors, resulting in higher frequencies of sexual intercourse (Rahman et al., 2005; Manning and Fink, 2008). These sexually selected traits are also often linked to higher levels of sex hormones such as testosterone and estradiol, which have been associated with increased sexual desire (van Anders, 2012; Cappelletti and Wallen, 2016) and higher sexual attractiveness (Giagulli et al., 2011; Haupt et al., 2020). Additionally, more masculine males and more feminine females reported higher levels of sexual satisfaction, consistent with previous studies (Pedersen and Blekesaune, 2003; Daniel and Bridges, 2013). Higher masculinity and femininity in males and females, respectively, may correlate with higher self-esteem and more positive body image, resulting in greater confidence and consequently, higher sexual satisfaction (Sánchez-Fuentes et al., 2014).

Findings from our study suggest that physical attractiveness and interpersonal relationships mediate the association between gender role conformity and sexual activity in males. As masculinity often correlates with physical attractiveness, men with more masculine appearances may tend to have better reproductive success. These results support the males-compete/females-choose (MCFC) model proposed by Darwin, suggesting that different sexes play different roles in reproduction, and that men typically compete for sexual attraction from females (Darwin, 2008; Dixson, 2020). Many other studies have also demonstrated that males have a stronger sense of intrasexual selection compared to females (Puts, 2010; Lassek and Gaulin, 2022). Physical attractiveness and interpersonal relationships constitute a part of the reproductive strategies of males; the former is utilized for short-term attractiveness (Buss et al., 2017; Buss and Schmitt, 2019), while the latter is utilized for long-term sexual relationships (Dixson et al., 2016). Studies have reported a female preference for more masculine craniofacial traits for short-term relationships than for long-term relationships (Little et al., 2011; Dixson et al., 2016). However, in long-term relationships, females prefer males with good moral or quasi-moral characteristics, such as intelligence, honesty, and warmth required for better interpersonal relationships (Regan et al., 2000). In addition, higher physical attractiveness and the ability to navigate interpersonal relationships are useful during courtship, crucial for reproductive success (Wong and Candolin, 2005; Eikenaar et al., 2012).

Another important finding was that sexual motivation had a greater mediating effect on females than on males. This is consistent with earlier observations. And the males-compete/females-choose model that females invest more in their offspring than their male counterparts became, in essence, a scarce resource, driving male–male competition to attract and retain mates. As mentioned above, females usually play the role of choosers in the mating process and have stronger internal motivation for intersexual selection (Eibl-Eibesfeldt, 1979; Stewart-Williams and Thomas, 2013).

Overall, this study has several strengths. First, it investigated the association between gender-role conformity and sexual activeness from a broader perspective by measuring not only sexual attitudes and experiences but also sexual behaviors and pleasure, allowing for a more precise evaluation of sexual activeness. Both masculinity and femininity were also accounted for. Second, mediation analysis was employed to investigate the associations of various factors with gender-role conformity through both biological and sociological pathways. This approach enabled us to better understand the gender-role patterns as they relate to sexual activeness between different sexes, increasing our understanding of sexual selection theory. Third, our study was based on a large-scale population-based survey in China. The large sample size ensured the statistical power of the modeling process, providing robust results with higher precision. More importantly, this study addresses the current knowledge gap concerning gender-role distribution among Chinese youths.

This study also has several limitations. First, as the measurement of gender-role conformity was self-reported, measurement bias may be present. However, as there has yet to be a robust gender-role measurement scale with high reliability and validity, this approach has the highest feasibility. Second, as the frequency of sexual intercourse was also self-reported, recall bias may be present. However, attempts were made to mitigate the severity of biasness by cross-checking survey questions and performing logic verification. Third, as this is a cross-sectional study, causal inference cannot be drawn. Fourth, the question “Do you want to have a girlfriend/boyfriend?” may not the best measure of sexual motivation. Intimate partnership establishment refers to both sexual and emotional intimacy, and someone who is highly motivated to have sex may prefer short-term relationship (i.e., casual sex, one-night stands) to avoid emotional intimacy. Finally, although we adjusted for possible confounders, we were unable to adjust for confounding from cultural and psychosocial factors, which may result in residual confounding. Future studies should consider these additional factors related to masculinity, femininity, and sexual activeness.

In conclusion, among heterosexual Chinese youths of reproductive age, high gender-role conformity (i.e., high masculinity in males and high femininity in females) was associated with a significantly higher level of sexual activeness, implying that androgynous tendencies may contribute to a decrease in population fertility. We also found that such an association between gender-role conformity and sexual activeness was mediated through physical attractiveness, sexual motivation, and interpersonal relationships.

## Data availability statement

The datasets presented in this article are not readily available because the data have not been made available on a permanent third-party archive because the Institution Review Board of Tsinghua University ruled that we could not post the data. Requests to access the datasets should be directed to the corresponding author (tangk@mail.tsinghua.edu.cn).

## Ethics statement

The study was reviewed and approved by the Institution Review Board of Tsinghua University (IRB no. 20190083). Written informed consent to participate in this study was provided by the participants.

## Author contributions

SZ and KT conceived of the presented idea. SZ and FG planned and carried out the simulations. SZ took the lead in writing the manuscript with the support from FG, JH, and KT. All authors contributed to the article and approved the submitted version.

## Conflict of interest

The authors declare that the research was conducted in the absence of any commercial or financial relationships that could be construed as a potential conflict of interest.

## Publisher’s note

All claims expressed in this article are solely those of the authors and do not necessarily represent those of their affiliated organizations, or those of the publisher, the editors and the reviewers. Any product that may be evaluated in this article, or claim that may be made by its manufacturer, is not guaranteed or endorsed by the publisher.
