# Supplementary material for: The mediating role of sexually selected traits in the association of androgynous tendencies with lower sexual activeness among Chinese youths
Source: Front Psychol. 2022 Oct 6;13:1011467. doi: 10.3389/fpsyg.2022.1011467 (PMC9583897; doi:10.3389/fpsyg.2022.1011467)
Supplement: Supplementary file 1 [file Data_Sheet_1.docx]

**Supplementary Information**

S1 Table. The associations between gender-role conformity score and mediators^a^.

|  |  |  | **Sex** | | |
| --- | --- | --- | --- | --- | --- |
| **Mediator** |  |  | **Male** |  | **Female** |
| ***Individual-level factors*** | |  |  |  |  |
| Self-perceived physical attractiveness (0-10)^b^ | |  | 0.49 (0.45-0.53)*** |  | 0.42 (0.40-0.44)*** |
| Eagerness to have intimate partners^c^ | |  | 1.07 (1.03-1.11)*** |  | 1.23 (1.20-1.26)*** |
| ***Interpersonal-level factors*** | |  |  |  |  |
| Self-rated interpersonal relationship (0-10)^b^ | |  | 0.55 (0.51-0.59)*** |  | 0.34 (0.32-0.36)*** |

The standardized gender-role conformity score was considered a continuous variable in all analyses, and the estimated coefficient (or odds) represented the change in outcome followed by a 1-SD change in SD.

1. We adjusted for age, region, ethnicity, school type, average monthly expenditure, self-rated parent–child relationship, having had received sexual education at school, having had parent–child discussions relevant to sexual behaviors, parents' highest educational attainments, tobacco consumption, and alcohol consumption.
2. We used a linear regression reporting coefficient and adjusted for age, region, ethnicity, school type, average monthly expenditure, self-rated parent–child relationship, having had received sexual education at school, parent–child discussion relevant to sexual behaviors, parents' highest educational attainments, tobacco consumption, and alcohol consumption.
3. We used logistic regression to report ORs, which were adjusted for age, region, ethnicity, school type, average monthly expenditure, self-rated parent–child relationship, having received sexual education at school, having had parent–child discussions relevant to sexual behaviors, parents' highest educational attainments, tobacco consumption, and alcohol consumption.

* *p* < 0.05, ** *p* < 0.01, and *** *p* < 0.001.

S2 Table. The statistical test of the exposure-mediator interaction effect

|  | *ACME (1) - ACME (0)* | *p-value* |
| --- | --- | --- |
| ***Individual-level factors*** | |  |
| Self-perceived physical attractiveness (0-10) | |  |
| Male | -0.0009 (-0.0002, 0.0000) | 0.046* |
| Female | -0.0005 (-0.0009, -0.0003) | < 0.001*** |
| Sexual motivation | |  |
| Male | 0.0000 (-0.0001, 0.0002) | 0.67 |
| Female | 0.0005 (0.0003, 0.0009) | < 0.001*** |
| ***Interpersonal-level factors*** | |  |
| Self-rated interpersonal relationship (0-10) | |  |
| Male | -0.0005 (-0.0014, 0.0006) | 0.388 |
| Female | -0.0008 (-0.0011, -0.0005) | < 0.001*** |

The standardized gender-role conformity score was considered a continuous variable in all analyses. p<0.05 meant that the exposure-mediator interaction was significant in the analysis, so the model included exposure-mediator interaction.

All models were adjusted for age, region, ethnicity, school type, average monthly expenditure, self-rated parent–child relationship, having had received sexual education at school, having had parent–child discussions relevant to sexual behaviors, parents' highest educational attainments, tobacco consumption, and alcohol consumption.

ACME = average causal mediation effect. ACME (1) considered the interaction effect between exposure and outcome, while ACME (0) did not.

* *p* < 0.05, ** *p* < 0.01, and *** *p* < 0.001.
